# The Role of Nrf2 in Liver Disease: Novel Molecular Mechanisms and Therapeutic Approaches

**DOI:** 10.3389/fphar.2018.01428

**Published:** 2019-01-08

**Authors:** Dongwei Xu, Min Xu, Seogsong Jeong, Yihan Qian, Hailong Wu, Qiang Xia, Xiaoni Kong

**Affiliations:** ^1^Department of Liver Surgery, Renji Hospital, School of Medicine, Shanghai Jiao Tong University, Shanghai, China; ^2^School of Pharmacy, Fudan University, Shanghai, China; ^3^Shanghai Key Laboratory for Molecular Imaging, Collaborative Research Center, Shanghai University of Medicine and Health Sciences, Shanghai, China

**Keywords:** Nrf2, oxidative stress, cytoprotective genes, acute liver injury, viral hepatitis, non-alcoholic fatty liver disease, non-alcoholic steatohepatitis, hepatocellular carcinoma

## Abstract

Oxidative stress and inflammation are the most important pathogenic events in the development and progression of liver diseases. Nuclear erythroid 2-related factor 2 (Nrf2) is the master regulator of the cellular protection *via* induction of anti-inflammatory, antioxidant, and cyto-protective genes expression. Multiple studies have shown that activation or suppression of this transcriptional factor significantly affect progression of liver diseases. Comprehensive understanding the roles of Nrf2 activation/expression and the outcomes of its activators/inhibitors are indispensable for defining the mechanisms and therapeutic strategies against liver diseases. In this current review, we discussed recent advances in the function and principal mechanisms by regulating Nrf2 in liver diseases, including acute liver failure, hepatic ischemia–reperfusion injury (IRI), alcoholic liver disease (ALD), viral hepatitis, non-alcoholic fatty liver disease (NAFLD), non-alcoholic steatohepatitis (NASH), and hepatocellular carcinoma (HCC).

## Introduction

Oxidative stress and inflammation are the most important pathogenic events in liver diseases. During liver injuries, the unregulated production of free radicals and/or ROS leads to damage of important biomolecules and cells and generation of proinflammatory genes. Antioxidant and anti-inflammatory therapy has been considered to be beneficial in liver diseases. Nrf2 is the master regulator of the primary means of cellular defense through mediation of antioxidant response, anti-inflammatory and cytoprotective properties, and dysregulation of Nrf2 activity has been revealed to correlated with the development of chronic inflammatory diseases (Alam et al., 1999; Vomund et al., 2017; Bellezza et al., 2018; Hennig et al., 2018). The protective effects of Nrf2 signaling pathway has been identified in a number of disease models, including acute kidney, lung or neurons injury, emphysema, and sepsis (Thimmulappa et al., 2007; Reddy et al., 2009; Sussan et al., 2009; Zhang et al., 2012; Liu et al., 2014). Accumulating evidence also has implicated this transcription factor in various liver diseases, including acute hepatoxicity, NAFLD, NASH, ALD, DILI, viral hepatitis, liver fibrosis, hepatic IRI, and primary hepatic malignancies (Klaassen and Reisman, 2010; Tang et al., 2014). Under acute and chronic oxidative stress and inflammatory conditions, Nrf2 is activated and prevents oxidative and inflammatory diseases by modulating genes expression of cytoprotective proteins and enzymes, which decreases ROS levels, inflammation, and cell death (Bataille and Manautou, 2012). However, the function of Nrf2 is not always protective in diseases, recent studies have identified that the gene expression of Nrf2 was associated with the pathogenesis, progression, and metastasis of cancer, resistance to cancer therapy, and the regulation of cancer cells metabolism, thereby suggesting that Nrf2 is a pleiotropic transcriptional factor (Karin and Dhar, 2016; Rojo de la Vega et al., 2018). In this review, we summarized up-to-date studies in the understanding of the roles and mechanisms of Nrf2 and the therapeutic approaches by targeting Nrf2 in liver diseases.

## Activation of Nrf2 Attenuates Acute Liver Injury

**FIGURE 1:**
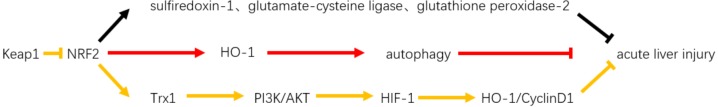
Role of Nrf2 in acute liver injury. The protective effects of Nrf2 in acute liver injury, one is through regulating antioxidant defense-related genes, including sulfiredoxin-1, glutamate-cysteine ligase, and glutathione peroxidase-2, and the other pathway is by promoting its target gene HO-1 and then enhanced autophagy. While its negative regulator-keap1, which by binding to it inhibits Nrf2 activation and Trx1-PI3K/AKT-HIF1-HO-1/CyclinD1 signal pathway and promotes liver injury.

Study has shown that activation of Nrf2 attenuates acute liver injury. Wu et al. (2012) compared serum ALT, LDH, hepatic hemorrhage, and necrosis levels between Nrf2-null and Nrf2-enhanced mice in cadmium-induced acute liver injury mice model; they found that Nrf2-enhanced mice were associated with lower ALT and LDH levels and with fewer morphological alterations. The mRNA levels of cytoprotective genes, including sulfiredoxin-1, glutamate-cysteine ligase, and glutathione peroxidase-2 were expressed only in Nrf2-enhanced mice, suggesting that Nrf2 activation prevents oxidative stress and acute liver injury through modulation of antioxidant defense-associated genes (Figure 1). Subsequently, the protective effects of Nrf2 was tested in LPS and D-GalN-induced liver injury mouse models by treatment with mangiferin, which could upregulate the gene expression of Nrf2 in a dose-dependent manner (Pan et al., 2016). Mangiferin treatment suppressed serum levels of ALT, AST, IL-1β, TNF-α, and ROS levels, adding evidences that activation of Nrf2 pathway protects against acute liver injury. Biochanin A, morin, curcumin, andrographolide, oxymatrine, and madecassoside were also found to play a protective role *via* activation of Nrf2 in LPS and D-GalN-induced acute liver injury in mice (Liu et al., 2016; Pan et al., 2017; Tian et al., 2017; Xie et al., 2017; Wang et al., 2018). In addition, the antioxidant pathway of Nrf2 was further tested and found to be effective in carbon tetrachloride-induced and acetaminophen-induced mouse acute liver injury models (Huang et al., 2016; Cao et al., 2017; Peng et al., 2018; Shen Z. et al., 2018). The role of Nrf2 in hepatic IRI was also identified by several studies (Ke et al., 2013; Kudoh et al., 2014; Rao et al., 2015; Ge et al., 2017; Xu et al., 2017). Ke et al. (2013) showed that the Keap1–Nrf2 complex could alleviate oxidative injury in mouse orthotopic liver transplantation through Keap1 signaling (Figure 1). The protective effects were identified by limiting hepatic inflammatory responses and hepatocellular necrosis. Recently, our research identified cytoprotective effects of CDDO-Im, a potent activator of the Nrf2 pathway, in hepatic IRI, through inducing Nrf2 target gene HO-1 expression leads to enhanced autophagy in hepatocytes, which results in increased clearance of damaged mitochondria, reduced mtDNA release and ROS production leading to reductions in DAMP release-induced inflammatory responses and subsequent secondary hepatocyte injury (Xu et al., 2017). Despite accumulating evidences, Nrf2-based treatment is yet to enter clinical trials in the USA ^1^ for patients with acute liver failure.

## Activation of Nrf2 Ameliorates Alcoholic Liver Disease

Alcohol consumption has been revealed to be significantly associated with the development and progression of liver diseases over decades (Shepard et al., 2010). Alcohol metabolism in the liver includes ethanol oxidation by alcohol dehydrogenase in hepatocytes and microsomal oxidation promoted by CYP2E1 (Bae et al., 2011; Wang et al., 2014a). Alcohol dehydrogenase-associated ethanol metabolism results in acetaldehyde, which gives rise to some downstream effects, such as depletion of glutathione, lipid peroxidation, and generation of ROS (Dey and Cederbaum, 2006). In addition, the dysregulation of antioxidant glutathione by Nrf2-dependent regulation was found to contribute to the development of ALD by providing pathological conditions, whereas the Nrf2-mediated antioxidant response provided protection against alcohol-induced oxidative stress by regulating glutathione metabolism (Harvey et al., 2009; Lu, 2013; Rejitha et al., 2015). Furthermore, the oxidative stress-induced upregulation of Nrf2 is considered to positively modulate expression of VLDLR, which contributes to ALD (Wang et al., 2014b).

**FIGURE 2:**
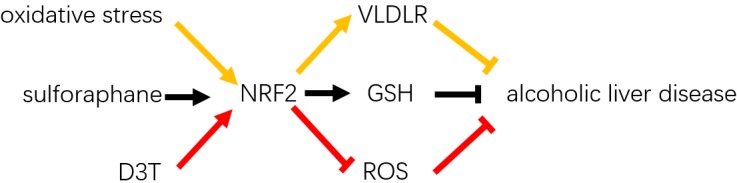
Role of Nrf2 in alcoholic liver disease. Studies shown that oxidative stress promotes Nrf2 activation-induced hepatic VLDLR overexpression and ameliorates ALD. Nrf2 activator-Sulforaphane protects alcoholic fatty liver disease by activating Nrf2 signaling pathway and GSH levels. D3T through upregulate Nrf2 protein levels and decreased ROS levels and liver injuries.

In ethanol-exposed mice, the role of Nrf2-induced antioxidant factors was first tested by the Nrf2 inducer D3T (Dong et al., 2008). Upregulation of Nrf2 by D3T treatment has significantly decreased generation of ethanol-induced ROS and apoptosis, which indicated that the activation of Nrf2 could diminish ethanol-induced apoptosis and ameliorate the disease status. Moreover, Zhou et al. (2014) verified that Nrf2-mediated cytoprotective enzymes could ameliorate alcohol-induced liver steatosis both in *in vivo* and *in vitro* models. They further administered sulforaphane, which is an activator of Nrf2 and present in considerable quantities in brassica vegetables including broccoli, cabbage, and kale, and found it to be effective in improving alcohol-induced liver steatosis (Figure 2). Furthermore, recent advances indicated that activation of the Nrf2 pathway was protective in alcohol-induced liver fibrosis and hepatotoxicity, whereas knockdown of Nrf2 was associated with enhanced alcohol-induced hepatocyte necroptosis (Song et al., 2015; Lu et al., 2016; Ni et al., 2017). By contrast, a more recent study demonstrated that ethyl pyruvate, which has multi-effects including antibacterial, anti-inflammatory, antiviral, vasodilatory, antioxidant, and antiapoptotic effects, decreases ALT, AST, hepatic morphological changes, triglycerides, free fatty acids, and the expression of proinflammatory factors and increases the expression of anti-inflammatory factors and peroxisome proliferator-activated receptor-α mRNA which through downregulation of the ROS–Nrf2 signaling pathway, thereby alleviating ALD in mice (Fawcett et al., 1999; Harding et al., 2000; Shen F. et al., 2018). Taken together, these evidences showed that Nrf2 activation plays essential protective role in the development of ALD and that simultaneous downregulation of Nrf2 with ROS and VLDLR may also be effective in the amelioration of ALD (Figure 2). Further studies are required to demonstrate the extent of amelioration between upregulation and downregulation of Nrf2 when ROS and VLDLR expression levels are downregulated in ALD.

## Protective Effects of Nrf2 on Viral Hepatitis-Infected Cells Against Oxidative Damage

**FIGURE 3:**
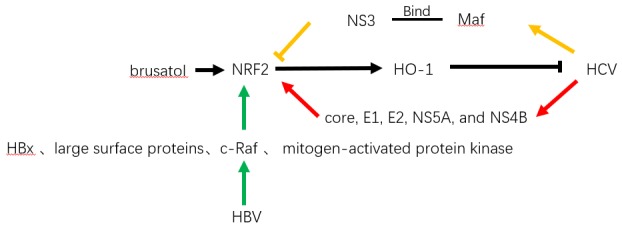
Role of Nrf2 in viral hepatitis-associated liver diseases. Hepatitis C virus was reported that impaired the protection of Nrf2 by promoting sMaf bound to NS3 and brusatol suppressed HCV infection by promoting Nrf2 pathway. HBV-regulatory proteins, including its X protein (HBx) and large surface proteins were reported to activate Nrf2 and antioxidative responses *via* c-Raf and mitogen-activated protein kinase.

Oxidative stress has been shown to be implicated in viral hepatitis-associated liver diseases, including HBV and HCV infections (Bolukbas et al., 2005; Ivanov et al., 2013). A previous study indicated that HCV could mediate the phosphorylation and activation of Nrf2, which was regulated by the mitogen-activated protein kinases. The authors further suggested that the activation of Nrf2-derived survival of HCV-infected cells may provide favorable circumstances for carcinogenesis (Burdette et al., 2010). Another study showed that the inhibition of Nrf2 and antioxidant response elements is regulated by the core proteins of HCV-replicating cell-triggered delocalization of small Maf proteins, which were bound to NS proteins NS3, thus reducing the expression of cytoprotective genes (Carvajal-Yepes et al., 2011). From the authors’ point of view, inhibition of Nrf2 and antioxidant response element-regulated genes may contribute to HCV-associated pathogenesis due to impaired induction of reactive oxygen intermediates caused by cytoprotective genes, which giving rise to host cell DNA damage and promoting the genetic variability of the viral genome. Moreover, Ivanov et al. (2011) found that the antioxidant-protective Nrf2/antioxidant response element pathway is activated by HCV proteins, including core, E1, E2, NS5A, and NS4B, in an ROS-dependent and -independent manners (Figure 3). In addition, a strong upregulation of the antioxidant-protective system was modulated in the earliest stage, indicating that Nrf2 is activated to protect against HCV-induced oxidative stress in the acute stage of HCV infection. In addition, replication of HCV has been reported to be suppressed by Nrf2-mediated heme oxygenase-1 (HO-1) inducible factor, which is a phytocompound isolated from *Lindera erythrocarpa* Makino fruits (lucidone), and a quinone methide triterpene isolated from *Tripterygium wilfordii* root extract (celastrol) (Chen et al., 2013; Tseng et al., 2017). Furthermore, an *in vitro* cell line study from Japan found that knockdown of Nrf2 significantly reduced HCV infection and steatosis (Sugiyama et al., 2014). Most recently, the authors further confirmed that an Nrf2 inhibitor (brusatol) had anti-HCV effects *in vitro* (Murakami et al., 2018) (Figure 3).

Hepatitis B virus infection, which causes acute or chronic liver inflammation and contributes to the development of HCC, has been shown to induce activation of Nrf2 and antioxidative response elements *in vivo* and *in vitro* by HBV-regulatory proteins, including HBx and large surface proteins, *via* c-Raf and mitogen-activated protein kinase (Hildt et al., 2002; Schaedler et al., 2010). In addition, the HBx protein-mediated activation of Nrf2 has been introduced to trigger the upregulation of glucose-6-phosphate dehydrogenase, thereby reprogramming metabolism of glucose, and may participate in the development of HCC (Liu et al., 2015). Therefore, Nrf2 is not only a crucial factor that is activated to defend against viral hepatitis-induced oxidative stress but also a protective factor that is involved in the survival of viral hepatitis-infected cells and may contribute to hepatocarcinogenesis.

## Protective Impact of Nrf2 in Non-Alcoholic Fatty Liver Disease

**FIGURE 4:**
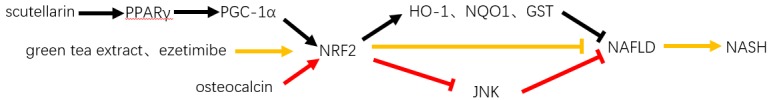
Role of Nrf2 in NAFLD. Multiple studies have shown that several pathways could activate Nrf2 and inhibit NAFLD and NASH including scutellarin-PPARγ/PGC1α-Nrf2-HO-1/NQO1/GST pathway; green tea extract and ezetimibe activated Nrf2 pathway; osteocalcin activated Nrf2 and subsequently inhibited JNK pathway.

Non-alcoholic fatty liver disease is a progressive disease arising from the accumulation of lipids in hepatocytes and has an increasing incidence worldwide (Satapathy and Sanyal, 2015). Approximately one-third of patients with NAFLD progress to severe NASH, which is linked with inflammation and cirrhosis (Tarantino and Finelli, 2013; Dietrich and Hellerbrand, 2014). Recent studies indicated that ROS and electrophiles are associated with the pathogenesis of NASH; thus, induction of Nrf2 seemed to be promising in the prevention and treatment of NAFLD (Chambel et al., 2015). Du et al. (2016), explored the therapeutic impact of Nrf2 activation by using osteocalcin, and found that it could improve NAFLD by ameliorating oxidative stress and inhibiting the JNK pathway, which is an important pathway involved in the pathogenesis of NAFLD. A recent study demonstrated that scutellarin, a flavonoid glycoside that has an antioxidative stress effect, significantly reduced blood lipid levels and enhanced the antioxidative capacity by activating PPARγ and its coactivator-1α, Nrf2, HO-1, GST, and NQO1, and suppressing nuclear factor κB and Keap1 at the mRNA and protein levels, thus ameliorating NAFLD (Zhang et al., 2018) (Figure 4). In addition, a modulator of PPARγ, apigenin, was also revealed to attenuate NAFLD by Nrf2-associated regulation of oxidative stress and hepatocyte lipid metabolism (Feng et al., 2017). Moreover, for the prevention of NAFLD, scutellarin, which is a natural drug with active components of breviscapine, was shown to be effective by enhancing the Nrf2-mediated antioxidant system in high-fat diet- and chronic stress-subjected rats (Fan et al., 2017).

Nuclear erythroid 2-related factor 2 has been found to be a key regulator in the protection against NASH (Gupte et al., 2013). By contrast, loss of Nrf2 or deletion of Nrf2 has been found to cause benign steatosis to develop into NASH and contribute to exacerbation of disease status (Chowdhry et al., 2010; Wang et al., 2013). Ramadori et al. (2017) indicated that overactivation of Nrf2 suppressed the hepatocyte-specific c-met deletion (an accelerative factor for NASH)-induced deleterious impact on the progression of NASH and suggested that Nrf2 repaired liver damage in hepatocyte-specific c-met-deficient mice *via* maintaining balance in cellular redox homeostasis. To date, green tea extract and ezetimibe (an inhibitor of Niemann-Pick-C1-Like 1) have been revealed to promote the protective impact of Nrf2 against lipid accumulation and the inflammatory response during NASH (Lee et al., 2016; Li et al., 2016) (Figure 4). However, Nrf2-associated therapeutic approaches for NASH remain to be implemented in a real-world clinical manner in the near future.

## Nrf2 in Primary Liver Cancer

**FIGURE 5:**
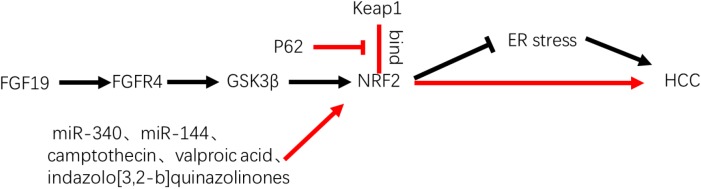
Role of Nrf2 in HCC. P62 and FGF19 were reported to activate Nrf2 and promote HCC by competing with Keap1 or through FGFR4–GSK3β signal pathway. MiR-340, miR-144, camptothecin, valproic acid, and indazolo[3,2-b]quinazolinones, which were revealed in suppressing Nrf2-dependent pathway and sensitizing HCC cells to anticancer treatments.

Hepatocellular carcinoma is the most common primary liver cancer, accounting for more than 80% of all hepatic malignancies (Forner et al., 2018), with molecular alterations in HCC arising in the very early stage of carcinogenesis (Pitot, 2007). Among the changes, activation of Nrf2 was found to be the prominent pathway that contributes to the progression of preneoplastic lesion to malignancy, which was confirmed by *in vivo* detection of the inhibition of the Nrf2 pathway that accompanied the regression of cytokeratin 19-positive nodules (Petrelli et al., 2014). The persistent activation of this transcription factor was found to be associated with the accumulation of p62, thus participating in the development of HCC (Inami et al., 2011). This finding was further supported by Saito et al. (2016) who confirmed the promotive impact of p62 in HCV-positive HCC through Nrf2-dependent metabolic reprogramming. In addition, Nrf2 was also found to participate in protection of HCC cells by facilitating the survival response of FGF19 to endoplasmic reticulum stress (Teng et al., 2017; Tian et al., 2018) (Figure 5). Thus, advances were made to regulate the Nrf2 pathway in HCC, including identification of miR-340, miR-144, camptothecin, and valproic acid, which were revealed to be effective in suppressing the Nrf2-dependent pathway, thereby sensitizing HCC cells to anticancer treatments (Shi et al., 2014; Zhou et al., 2016; Chen et al., 2017; Yu et al., 2017) (Figure 5). Moreover, indazolo[3,2-b]quinazolinones were revealed to attack HCC cells by suppressing Nrf2/antioxidative response elements and inducing mitochondrial-dependent apoptosis simultaneously (Zhang et al., 2016). In a clinical retrospective study, patients with high expression levels of Nrf2 (*n* = 48) had significantly reduced overall (median, 13.87 months) and disease-free survival (median, 11.24 months) compared with patients with low expression levels of Nrf2 (*n* = 17), who exhibited median overall survival of 30.40 months and disease-free survival of 24.43 months (P < 0.01) (Zhang et al., 2015). The relative risk of high Nrf2 levels in overall survival was 5.96 with 95% confidence interval of 2.46–14.69 (*P* < 0.01). However, regarding the sample size and retrospective nature, a large-sized prospective clinical study is required to confirm the prognostic impact of Nrf2 in patients with HCC.

## Conclusion

In this review, we briefly summarized the biology characteristics of Nrf2 pathway and discussed the potential therapeutic applications of targeting Nrf2 in liver diseases. To date, there are currently few pharmacological options available to prevent or treat liver diseases. Recently, in clinical trial, NGM282, an engineered FGF19 analog, could significantly reduce liver fat content in patients with NASH and remarkably improve ALP and transaminase levels in patients with PBC (Harrison et al., 2018; Mayo et al., 2018). The small molecule PRI-724 also identified the anti-fibrotic effects in a phase 1 trial in patients with HCV cirrhosis (Kudo et al., 2018).

A link between liver diseases and oxidative stress is indispensable. The Nrf2 antioxidant pathway is activated to protect the liver by modulating defensive genes, which even protect viral hepatitis-infected cells and HCC cells. A number of preclinical studies have detected regulatory factors for Nrf2; however, further identification of Nrf2 activators for liver injury/failure and Nrf2 inhibitors for viral hepatitis, and HCC is promising for the establishment of extensive and effective approaches to improve the prognosis of liver diseases. Regarding the great potential of this transcription factor, there is an unmet need for prospective clinical trials to explore the therapeutic impact of Nrf2 regulation in patients with liver diseases.

## Author Contributions

DX and MX wrote the manuscript. SJ and YQ wrote some part of the manuscript and made language retouching for our manuscript. HW revised the manuscript. QX and XK designed and revised the manuscript.

## Conflict of Interest Statement

The authors declare that the research was conducted in the absence of any commercial or financial relationships that could be construed as a potential conflict of interest.

## Footnotes

1 clinicaltrials.gov

## Abbreviations

ALD: alcoholic liver disease
ALP: alkaline phosphatase
ALT: alanine aminotransferase
AST: aspartate aminotransferase
CDDO-Im: CDDO-imidazolide
CYP2E1: cytochrome P450 2E1
D3T: 3H-1,2 dithiole-3-thione
D-GalN: D-galactosamine
DAMP: damage-associated molecular pattern
DILI: drug-induced liver injury
FGF19: fibroblast growth factor 19
GST: glutathione S-transferase
HBV: hepatitis B virus
HBx: HBV stimulates by its X protein
HCC: hepatocellular carcinoma
HCV: hepatitis C virus
IRI: ischemia–reperfusion injury
JNK: c-Jun N-terminal kinase
Keap1: Kelch-like ECH-associated protein
LDH: lactate dehydrogenase
LPS: lipopolysaccharide
NAFLD: non-alcoholic fatty liver disease
NASH: non-alcoholic steatohepatitis
NQO1: NAD(P)H quinone dehydrogenase one
Nrf2: nuclear erythroid 2-related factor 2
NS: non-structural
PBC: primary biliary cholangitis
PPARγ: peroxisome proliferator-activated receptor-γ
ROS: reactive oxygen species
VLDLR: very-low density lipoprotein receptor
